# Osteosarcoma in One of Identical Twins: Three Cases Report and a Literature Review

**DOI:** 10.1111/os.13004

**Published:** 2021-05-05

**Authors:** Jie Zhao, Wei Wang, Zhiyong Liu, Xiao Li, Qiqing Cai, Xiuchun Yu

**Affiliations:** ^1^ Department of Orthopaedic Surgery The Affiliated Hospital of Shandong University of Traditional Chinese Medicine Jinan China; ^2^ Department of Orthopaedic Surgery The 960th Hospital of the PLA Joint Logistics Support Force Jinan China; ^3^ Department of Pediatric Orthopedics Linyi People's Hospital Linyi China; ^4^ Bone and Soft Department The Affiliated Cancer Hospital of Zheng Zhou University, He Nan Cancer Hospital Zhengzhou China

**Keywords:** clinical characteristics, genetic variants, identical twins, osteosarcoma, siblings

## Abstract

**Background:**

Osteosarcoma (OS) is the most common primary malignant bone tumor occurring mainly in children and young adults. OS is usually seen in sporadic cases, and it is an extremely rare phenomenon in blood relatives, particularly among identical twins.

**Case Presentation:**

The present study reports three cases of OS occurring in only one of identical twins. The first case is a high‐grade OS in the left proximal tibia of a 16‐year‐old girl, treated with neo‐adjuvant chemotherapy, en bloc resection, and reconstruction with a modular knee tumor prosthesis. The second one is a high‐grade OS of the left proximal tibia of a 6‐year‐old girl. The patient was treated with neo‐adjuvant chemotherapy, en bloc resection, and reconstruction with inactived autograft. The third one is a conventional OS of the right proximal tibia of a 20‐year‐old woman. She was treated with neo‐adjuvant chemotherapy, en bloc resection, and reconstruction with a custom‐made prosthesis.

**Conclusions:**

The occurrence of OS in one of identical twins is a relatively rare event but may present the best opportunity to understand the genetic mechanisms underlying the tumorigenesis and progression of this disease in humans. A longer follow‐up period and regular imaging evaluation are needed to confirm whether the identical twin of these patients will suffer OS in the future.

## Graphical abstract

Osteosarcoma is rare in identical twins. We report three osteosarcoma patients whose sibling is healthy and review the clinical characteristics of 42 osteosarcoma patients who have siblings described in previous literature. Comparison of pathogenic mutations between osteosarcoma patients and their healthy twin may contribute to the discovery of novel therapeutic targets.

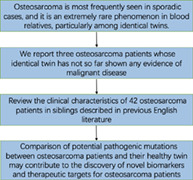

## Introduction

Osteosarcoma (OS) is one of the most common primary bone malignancies in children and adolescents^1^. OS accounts for 3.4% of pediatric tumors and 20% of primary bone cancers^2^. Nonetheless, it is a relatively rare neoplasia, with an incidence of 400–600 cases per year in the United States^3^, ^4^. Most OS cases are usually sporadic in nature with no positive family history or identifiable predisposing factors. The risk of OS is increased in patients with various cancer predisposition syndromes, including hereditary retinoblastoma, Li–Fraumeni syndrome, Rothmund–Thomson syndrome, and Bloom syndrome^5^, ^6^, ^7^. OS is an even rarer phenomenon in siblings, occurring in fewer than 10 in 10,000 patients^8^, ^9^.To the best of the authors’ knowledge, there is only one report of OS affecting identical twins in the relevant English literature^4^.

We report three OS patients whose identical twin has not so far shown any evidence of malignant disease (Table 1). The first case is a high‐grade OS in the left proximal tibia of a 16‐year‐old girl, treated with neo‐adjuvant chemotherapy, en bloc resection, and reconstruction with a modular knee tumor prosthesis. The second case is a high‐grade OS of the left proximal tibia of a 6‐year‐old girl. The patient was treated with neoadjuvant chemotherapy, en bloc resection, and reconstruction with inactived autograft. The third case is a conventional OS of the right proximal tibia of a 20‐year‐old girl. She was treated with neoadjuvant chemotherapy, en bloc resection, and reconstruction with a custom‐made prosthesis. We also review the clinical characteristics of 42 OS patients who have siblings described in detail in previous English literature (Table 2).

**TABLE 1:** General data of three osteosarcoma patients in three pairs of identical twins

Case	Sex/age (years)	Site	Race	Past history	Histologic subtype	MSTS stage	Treatment	Follow‐up (months)	Replase	Outcome
1	F/16	LPT	Chinese	None	OOS	IIB	NCT + Resection + CT	36	Pulmonary metastasis	Died
2	F/6	LPT	Chinese	None	COS	IIB	NCT + Resection + CT	48	None	Alive
3	F/20	RPT	Chinese	None	FOS	IIB	NCT + Resection + CT	10	Pulmonary metastasis	Died

COS, chondroblastic osteosarcoma; CT, chemotherapy; FOS, fibroblastic osteosarcoma; LPT, left proximal tibia; MSTS, musculoskeletal tumor society; NCT, Neoadjuvant chemotherapy; OOS, osteoblastic osteosarcoma; RPT, right proximal tibia.

**TABLE 2:** Osteosarcoma in siblings: review of the English literature

No.	Report	Relationships	Sex/age (years)	Site	Race	Past history	Histologic subtype	Molecular/genetic abnormality	Treatment	Follow‐up (months)	Relapse	Outcome	Refs.
1	Roberts CW, *et al*. (1935)	3 siblings	M/23	RPT	Caucasian	None	RCO	Unknown	None	3	Pulmonary metastasis	Died	^10^
2			F/13	RPH	Caucasian	None	RCO	Unknown	Radiation	4	None	Died	
3			F/17	RDF	Caucasian	None	RCO	Unknown	Amputation	8	Pulmonary metastasis	Died	
4	Pohle EA, *et al*. (1936)	2 sisters	F/3	RDF	American	None	COS	Unknown	Amputation/radiation	8	Pulmonary metastasis	Unknown	^11^
5			F/11	RDU	American	None	OOS	Unknown	Amputation/radiation	1	Unknown	Unknown	
6	Barry HC. (1961)	2 brothers	M/55	LDH	Australian	Paget's disease	Secondary	Unknown	Radiation	10	Unknown	Died	^12^
7			M/53	Sacrum	Australian	Paget's disease	Secondary	Unknown	Resection	6	Unknown	Died	
8	Harmon TP, *et al*. (1966)	4 siblings	M/15	RDF	Unknown	None	OOS	Unknown	Amputation	10	Pulmonary metastasis	Died	^13^
9			M/20	LDT	Unknown	None	OOS	Unknown	Amputation	192	None	Alive	
10			F/11	LPT	Unknown	None	OOS	Unknown	Radiation	96	None	Alive	
11			M/22	LPT	Unknown	None	FOS	Unknown	Amputation	18	Pulmonary metastasis	Died	
12	Swaney JJ. (1973)	2 brothers	M/11	RPF	Unknown	None	Unknown	Unknown	Hemipelvectomy/ chemotherapy	6	Pulmonary metastasis	Alive	^14^
13			M/4	LPT	Unknown	None	Unknown	Unknown	Radiation/amputation	8	Pulmonary metastasis	Died	
14	Schimke RN, *et al*. (1974)	2 siblings	F/11	RDF	Unknown	Bilateral RB, radiation	Unknown	Germline RB1 mutation	Amputation	6	Local recurrence, pulmonary metastasis	Died	^8^
15			M/9	RDF	Unknown	Bilateral RB, radiation	Unknown	Germline RB1 mutation	Radiation	2	Pulmonary metastasis	Died	
16	Mulvihill JJ, *et al*. (1977)	3 siblings	F/15	LPT	American Indian	None	OOS	Chromosomal breaks/HLA phenotypes	Radiation /amputation	79	None	Alive	^15^
17			F/7	RDF	American Indian	Limb anomaly	OOS	Unknown	Radiation/amputation	28	Bone metastasis	Died	
18			M/18	RPT	American Indian	None	OOS	Chromosomal breaks/HLA phenotypes	Amputation	10	Pulmonary metastasis	Died	
19	Miller CW, *et al*. (1977)	2 sisters	F/17	RDF	African American	None	Unknown	Unknown	Amputation/chemotherapy	12	None	Alive	^16^
20			F/15	RDF	African American	None	Unknown	Unknown	Amputation	24	Metastasis (lung, liver)	Died	
21	Colyer RA, *et al*. (1979)	2 siblings	F/16	LPH	Unknown	None	Unknown	Unknown	Amputation/chemotherapy	23	Pulmonary metastasis	Died	^17^
22			M/11	RDF	Unknown	None	Unknown	Unknown	None	8	Pulmonary metastasis	Died	
23	Brenton DP, *et al*. (1980)	2 brothers	M/57	Left pelvis	Unknown	Paget's disease	Secondary	Unknown	Radiation/chemotherapy	4	Pulmonary metastasis	Died	^18^
24			M/55	RDF	Unknown	Paget's disease	Secondary	Unknown	Unknown	Unknown	Unknown	Died	
25	Gilman PA, *et al*. (1985)	2 sisters	F/8	RPF	American‐Indian	None	Unknown	Chr 13;14 Rearrangement	Chemotherapy/resection	48	Pulmonary metastasis	Alive	^19^
26			F/12	RDF	American‐Indian	None	Unknown	Chr 13;14 Rearrangement	Amputation	13	Pulmonary metastasis	Died	
27	Hillmann A, *et al*. (2000)	2 siblings	F/11	RDF	Caucasian	None	OOS	Unknown	Chemotherapy/amputation	108	None	Alive	^20^
28			M/14	LDF^§^	Caucasian	None	OOS	No abnormality of TP53 and RB1	Chemotherapy/resection	48	None	Alive	
29	Shinozaki T, *et al*. (2000)	2 siblings	F/12	LDF	Japanese	None	OOS	HLA phenotypes	Amputation/chemotherapy	48	None	Alive	^21^
30			M/18	LDF	Japanese	None	PDOS	HLA phenotypes	Amputation/chemotherapy	72	None	Alive	
31	Chin KR, *et al*. (2001)	2 brothers	M/18	LDF	African American	None	OOS	Unknown	Chemotherapy/resection	36	Metastasis (lung, spine)	Died	^22^
32			M/21	RDF	African American	Tobacco	COS	No deletion of TP53 and RB1	Chemotherapy/Resection	Unknown	None	Died	
33	Longhi A, *et al*. (2001)	2 brothers	M/15	LDH	Unknown	Unknown	OOS	C‐myc, c‐fos, Cdk4 overexpression	Amputation/chemotherapy	36	Metastasis (tibia, lung)	Died	^9^
34			M/21	LPH	Unknown	Unknown	COS	Cdk4, MDM2 overexpression	Chemotherapy/resection	48	None	Alive	
35	Ottaviani G, *et al*. (2002)	2 siblings	F/11	LDF	Caucasian	None	TOS	Unknown	Chemotherapy/resection	204	Recurrence	Alive	^23^
36			M/12	BNR	Caucasian	None	TOS	Unknown	Chemotherapy/resection	48	None	Alive	
37	Biazzo A, *et al*. (2014)	2 identical twins	M/25	RPT	Unknown	None	POS	Unknown	Resection	96	None	Alive	^4^
38			M/33	LDT	Unknown	None	Unknown	Unknown	Chemotherapy/resection	12	None	Alive	
39	Ji JL, *et al*. (2017)	2 brothers	M/22	BPT^#^	Caucasian	ATR‐X syndrome	FOS	Germline ATRX mutation,13q deletion, 17p gain	Bilateral amputation	60	Pulmonary metastasis	Died	^24^
40			M/22	RPF	Caucasian	ATR‐X syndrome	EOS	Germline ATRX mutation; LOH of RB1 and TP53	Amputation	12	Pulmonary metastasis	Died	
41	Colombo EA, *et al*. (2018)	2 siblings	F/23	Olecranon	Caucasian	RTS	OOS	RECQL4 mutation	Chemotherapy	Unknown	None	Died	^25^
42			M/19	RDT	Caucasian	RTS	FOS	RECQL4 mutation	Amputation	Unknown	Bone metastasis	Alive	

BNR, bilateral ninth rib; BPT^#^, bilateral proximal tibia (two metachronous tumors); COS, chondroblastic osteosarcoma; EOS, epithelioid osteosarcoma; FOS, fibroblastic osteosarcoma; HLA, human leukocyte antigen; LDF, left distal femur; LDF^§^, left distal fibula; LDT, left distal tibia; LDH, left distal humerus; LOH, loss of heterozygosity; LPH, left proximal humerus; LPT, left proximal tibia; OOS, Osteoblastic osteosarcoma; PDOS, poorly differentiated osteosarcoma; POS, parosteal osteosarcoma; RB, retinoblastoma; RCO, Round‐cell osteosarcoma; RDF, right distal femur; RDT, right distal tibia; RDU, right distal ulna; RPF, right proximal femur; RPH, right proximal humerus; RPT, right proximal tibia; RTS, Rothmund–Thomson Syndrome; TOS, telangiectatic osteosarcoma.

## Case Report

### Case 1

**Fig 1:**
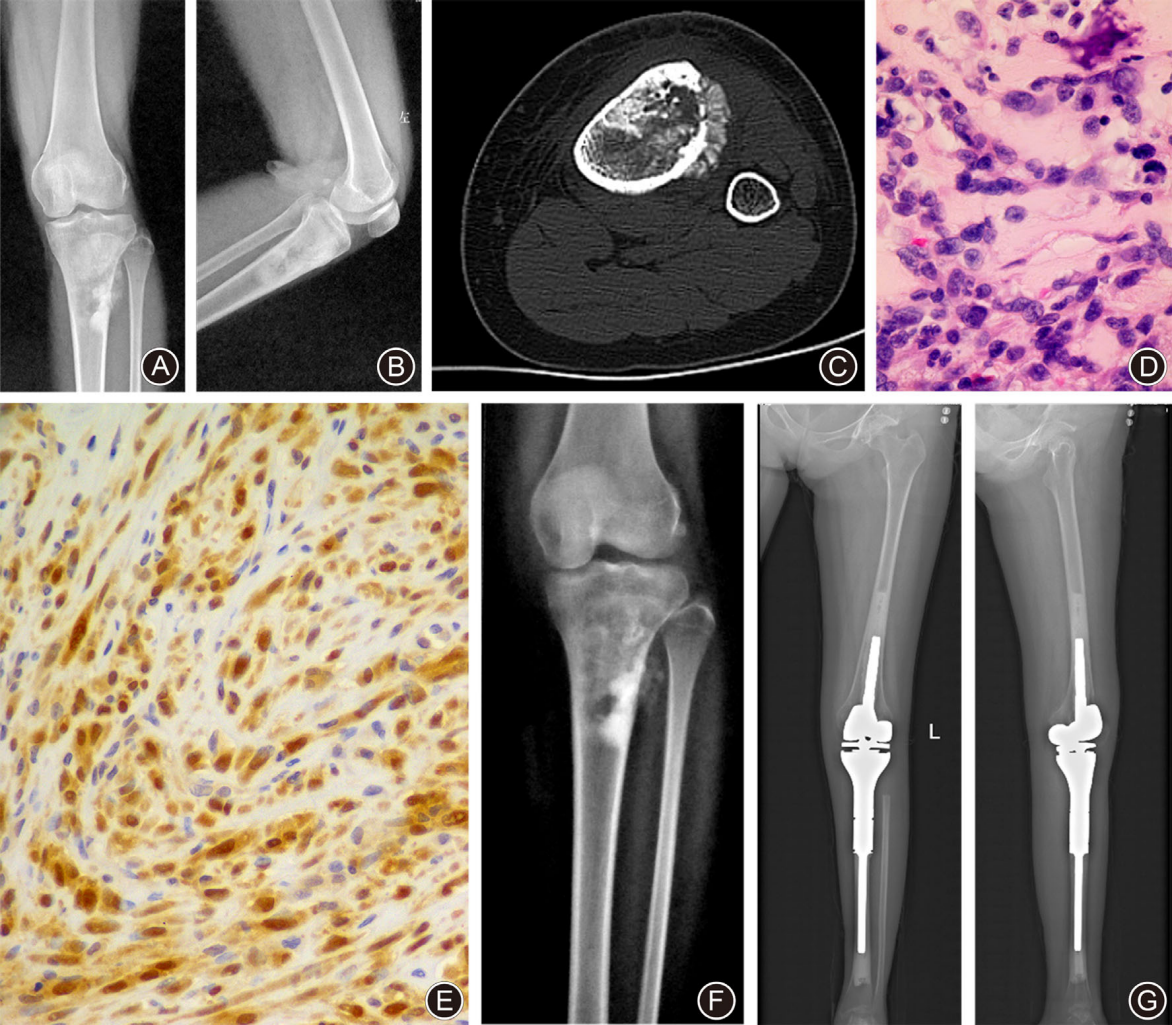
Female, 16 years old, left proximal tibial osteosarcoma. (A, B) Anteroposterior and lateral plain radiographs of the left knee showed a mixed lesion in the metaphysis of the left proximal tibia, with periosteal reaction and soft tissue mass. (C) CT axial imaging of the proximal tibia showed tumor bone formation in the medullary cavity, cortical penetration, and soft tissue mass. (D) Percutaneous needle biopsy of the left proximal tibia was interpreted as a high‐grade osteoblastic osteosarcoma. HE staining (×400) showed that there were a large number of spindle‐shaped cells with mild nuclear atypia. Neoplastic woven bones were also seen. (E) The immunohistochemical analysis showed that the p16 protein was present in more than 50% of tumor cells (×200). (F) After chemotherapy, there was apparent tumor calcification in the anteroposterior plain radiograph of the left knee. (G, H) X‐rays showed knee tumor prosthesis was in good position at the last follow‐up.

In June 2017, a 16‐year‐old Chinese girl, whose younger identical twin sister was healthy, was referred to our institute for complaints of left knee pain of approximately 3‐months duration. Her past history was negative for trauma, infection, irradiation, or cancer. Anteroposterior (Fig. 1A) and lateral (Fig. 1B) plain radiographs of the left lower extremity taken at an external hospital revealed mixed destruction of osteolytic and osteogenic focus in the metaphysis of the left proximal tibia, with periosteal reaction and soft tissue mass. The patient was admitted to our hospital for further examination and treatment. On physical examination, the findings were an obvious tender and slightly swollen area on the anterolateral aspect of the left proximal tibia with normal temperature and color, and both knees had full and symmetric range of motion (ROM).

Chest X‐ray and computed tomography (CT) scans did not show any evidence of pulmonary metastases. Emission computerized tomography (ECT) was positive for the lesion in the left proximal tibia, but not elsewhere. The axial CT image (Fig. 1C) of the left lower extremity showed tumor bone formation in the medullary cavity, cortical penetration, and soft tissue mass. Percutaneous needle biopsy of the left proximal tibia was interpreted as an osteoblastic osteosarcoma (Fig. 1D).The immunohistochemical (IHC) analysis showed that the p16 protein was present in more than 50% of tumor cells and a strong positive vimentin expression was detected (Fig. 1E).The patient received neoadjuvant chemotherapy consisting of cisplatin (120 mg/m^2^), ifosfamide (2 g/m^2^), and doxorubicin (75 mg/m^2^) for two cycles. After chemotherapy, there was apparent tumor calcification in the anteroposterior (Fig. 1F) plain radiograph of the left lower extremity. She was staged as IIB according to the Musculoskeletal Tumor Society (MSTS) staging system. On 4 August 2017, she underwent wide intra‐articular resection of the left proximal tibia and reconstruction with a cemented, modular, rotating‐hinge tumor knee prosthesis (Wego, Beijing, China) (Fig. 1G,H). The surgical margins of resected specimens were negative for tumor. The patient also completed six courses of postoperative chemotherapy without any complication. However, pulmonary metastases developed within 9 months after surgery. She was treated with three‐dimensional conformal radiotherapy (3‐DCRT) to a total dose of 20 Gy in six fractions. After radiotherapy, she was found to have new small pulmonary nodules. Then she received apatinib, a novel oral small‐molecule tyrosine kinase inhibitor (TKI) targeting the intracellular domain of vascular endothelial growth factor receptor‐2 (VEGFR‐2). Unfortunately, the treatment failed. The patient died of spontaneous pneumothorax 3 years after initial diagnosis.

### Case 2

**Fig 2:**
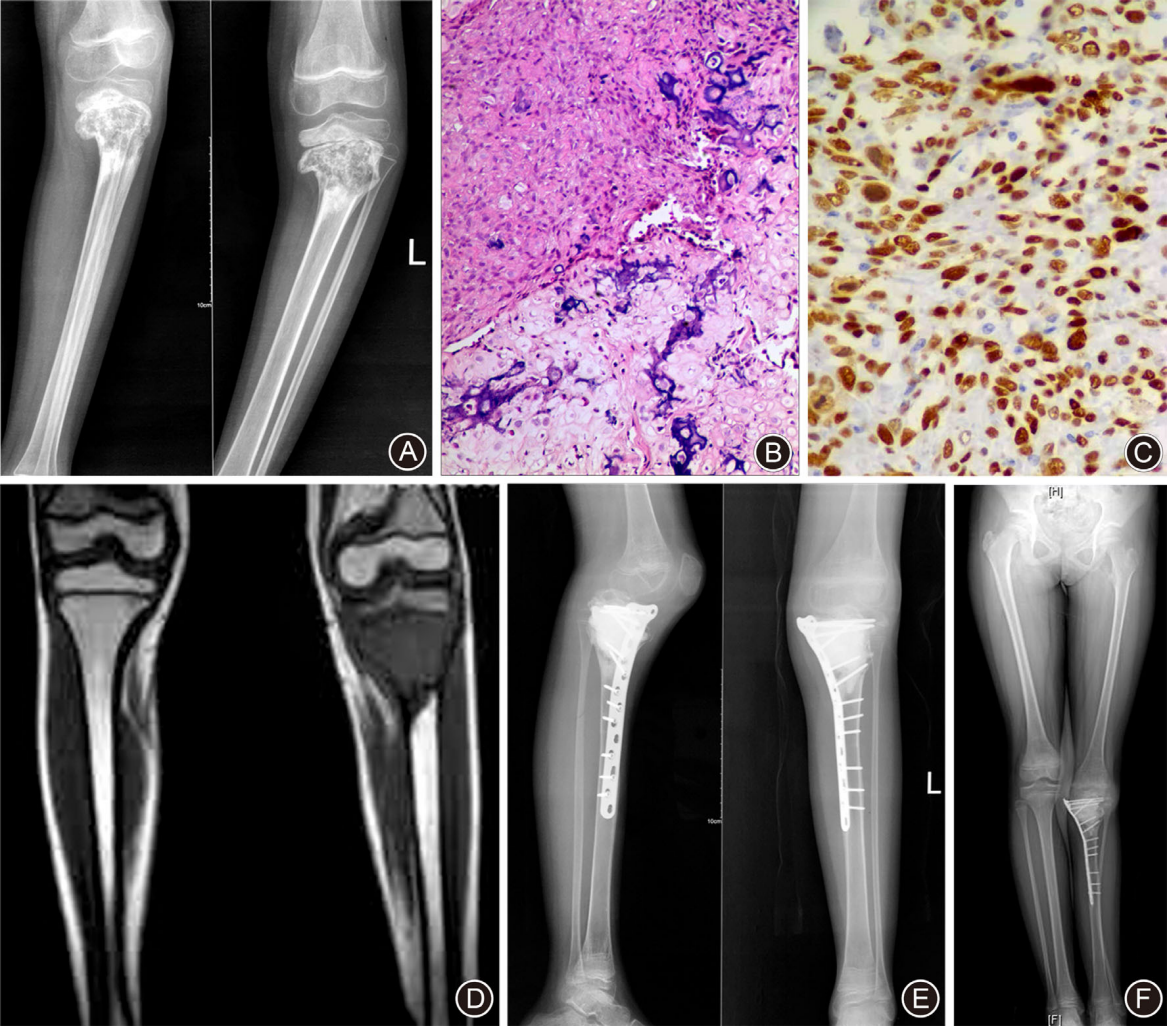
Female, 6 years old, left proximal tibial osteosarcoma. (A) The anteroposterior and lateral X‐rays before chemotherapy showed osteolytic lesion in the metaphysis of left proximal tibia. (B) Histological examination of the biopsy specimen demonstrated chondroblastic OS. Most tumor cells were spindle‐shaped with moderately heteromorphic nuclei. These cells produced osteoid describing irregular trabeculae with central calcification. Occasionally, macronucleoli and multinucleated giant cells were found. Mitotic figures were common. (HE stain, ×100). (C) IHC analysis showed that p53 protein was present in more than 40% of tumor cells. (×200). (D) The MRI images after chemotherapy showed a destructive lesion of the proximal tibia extending from the metaphysis to the epiphysis beyond the epiphyseal line or plate. (E) Radiographs taken 1 year after surgery showed good bone union at the graft‐host junction and mild posterior dislocation of the knee joint. (F) The X‐ray radiography for whole low extremities showed leg length discrepancy.

In June 2016, a 6‐year‐old Chinese girl was admitted to our hospital with 2‐months history of left knee pain. No history of trauma, infection, irradiation, or cancer was found. The anteroposterior and lateral radiographs (Fig. 2A) before chemotherapy showed osteolytic lesion in the metaphysis of left proximal tibia. Bone scans were negative for bone metastasis. No definite metastatic nodules were found in CT images of the lungs. A core needle biopsy was performed with diagnosis of high‐grade chondroblastic OS (Fig. 2B). p53 protein accumulation was seen in 40% of tumor cells and S‐100 expression was seen in 25% of tumor cells (Fig. 2C). After two cycles of neoadjuvant chemotherapy, the magnetic resonance imaging (MRI) (Fig. 2D) showed a destructive lesion of the proximal tibia extending from the metaphysis to the epiphysis beyond the epiphyseal line or plate. Then she underwent wide tumor resection and reconstruction of the intercalary bone defect with alcohol‐induced devitalized bone segment and plate. Then she completed 10 cycles of postoperative adjuvant chemotherapy. Radiographs (Fig. 2E) taken 1 year after operation demonstrated good bone union at the graft–host junction. However, surgical‐related complications including posterior knee dislocation (Fig. 2E) and lower limb discrepancy (Fig. 2F) were identified. At last follow‐up in September 2020, the patient was continuously disease‐free and without functional deficits. Her identical twin sister remains healthy through the follow‐up period.

### Case 3

**Fig 3:**
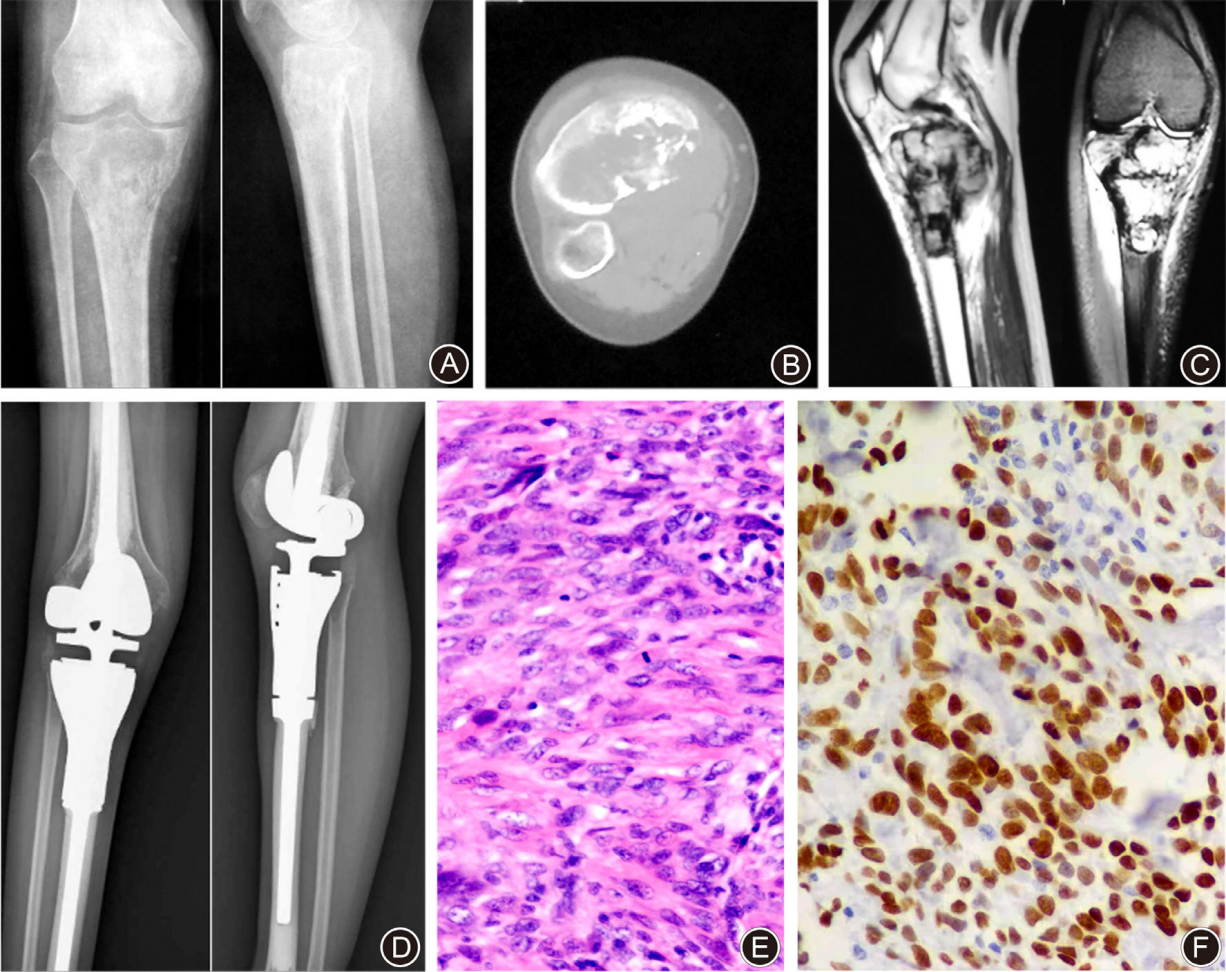
Female, 20 years old, right proximal tibial osteosarcoma. (A) Preoperative X‐rays showed an osteolytic lesion with high‐density tumor bone in the metaphysis of the right proximal tibia. (B) Axial CT image showed irregular bony destruction and extraosseous soft tissue mass. (C) MRI showed a tumor with low signal intensity on sagittal T1WI and high signal intensity on coronal T2WI with evidence of a posterior soft tissue mass. (D) After wide resection of bone tumor, a modular knee tumor prosthesis was implanted (Wego, Beijing, China). (E) Photomicrograph of the surgical specimen (HE staining, ×100). The tumor cells were spindle‐shaped with large deep‐stained nucleus, and a small amount of osteoid deposition was seen. (F) A strong SATB‐2 expression was seen in the majority of tumor cells (×200).

A 20‐year‐old Chinese female, with no family history of malignant tumors or irradiation, fell and hurt her right knee in physical education class in school in January 2018. Since that time, she experienced constant pain in the proximal leg for 3 months. X‐ray films of her right knee (Fig. 3A) revealed a destructive lesion at the proximal tibia with soft tissue extension. Axial CT image (Fig. 3B) showed tumor new bone formation and cortical discontinuity on medial aspect. MRI (Fig. 3C) showed a tumor with low signal intensity on sagittal T1‐weighted imaging (T1WI) and high signal intensity on coronal T2‐weighted imaging (T2WI) with evidence of a posterior soft tissue mass. No metastatic lesions were seen on CT scans of the chest and abdomen. A biopsy of the lesion was done on 29 April 2018 and a diagnosis of primary conventional OS was made. The patient was started on two cycles of DIA neoadjuvant chemotherapy. Then she underwent wide resection of bone sarcoma and modular knee tumor prosthetic replacement (Wego, Beijing, China) (Fig. 3D). The postoperative pathological analysis confirmed the diagnosis of high‐grade fibroblastic OS (Fig. 3E). Strong positivity for SATB‐2 protein was seen in the majority of tumor cells by IHC (Fig. 3F). This was followed by postoperative adjuvant chemotherapy. However, the patient died of respiratory failure due to advanced pulmonary metastasis in October 2019.

## Discussion

In 1935, Roberts and Roberts firstly reported the concurrent development of osteogenic sarcoma in three siblings^10^. Since then, including a pair of identical twins, 42 OS patients in 19 families have been reported on in detail^4^, ^8^, ^9^, ^11^, ^12^, ^13^, ^14^, ^15^, ^16^, ^17^, ^18^, ^19^, ^20^, ^21^, ^22^, ^23^, ^24^, ^25^. We collected the clinical, pathological, and molecular characteristics of 42 cases, including blood relationships, sex, age, race, past history, tumor location, histopathological diagnosis, genetic abnormality, treatment, follow‐up results. The complete data are shown in Table 2. In these studies, 16 out of 19 reports were two siblings (84.2%), two reports (10.5%) were three siblings, and only one report (5.3%) was four siblings. There were 25 males and 17 females (male/female: 1.47). The mean age at initial diagnosis was 19 ± 13 years old (range: 3–57 years). The anatomic locations of the tumors included distal femur (17 cases, 40.5%), proximal tibia (eight cases, 19%), proximal femur (three cases, 7.1%), proximal humerus (three cases, 7.1%), distal tibia (three cases, 7.1%), distal humerus (two cases, 4.8%), fibula, distal ulna, sacrum, pelvis, rib, and olecranon (one case for each site). This distribution pattern was almost the same as that reported in a population‐based study using data from the National Cancer Institute's Surveillance, Epidemiology, and End Results (SEER) program^26^. Twenty‐six percent of the patients were Caucasian, 12% were American Indian, 9.5% were African American, and 38% were unknown or unreported. In 40 of 42 cases (95%), previous medical history was noted. Most patients (28 cases, 66.7%) were negative for past musculoskeletal disorders. Four patients had a history of Paget's disease, two had ATR‐X syndrome, two had bilateral retinoblastomas and radiation exposure, two had Rothmund–Thomson Syndrome, one had a metachronous OS on the contralateral side, and one had an anomaly of the limb^8^, ^12^, ^18^, ^24^, ^25^. The main histological subtypes of OS were osteoblastic (12 cases, 28.6%), chondroblastic (three cases, 7.1%), and fibroblastic (three cases, 7.1%), with rare subtypes including secondary (four cases, 9.5%), small round‐cell (three cases, 7.1%), telangiectatic (two cases, 4.8%), parosteal (one case, 2.4%), and epithelioid OS (one case, 2.4%). Before the 1970s, amputation (6/11, 54.5%) and radiotherapy (5/11, 45.5%) were the major treatments for patients with non‐metastatic OS and 5‐year survival rate was only 18%. Despite the introduction and use of chemotherapy to the treatment in the 1970s, long‐term survival rate for OS patients in siblings was not significantly improved, which was less than that reported in the sporadic osteosarcomas^27^, ^28^. During a median follow‐up time of 15.5 months (range: 1–204 months), pulmonary metastasis was found in about 47.6% of patients, which was significantly higher than that in sporadic OS cases^29^. There were 16 patients (38%) who survived and 24 patients (57%) who died. In our reports, pulmonary metastasis occurred in two cases (2/3, 66.7%) and they finally died.

Pediatric OS is characterized by multiple somatic chromosomal lesions, including structural variations and copy number alterations (CNAs)^30^. The OS genome has long been known to be complex and heterogeneous, with few common features between tumors. Previously, various somatic mutations and copy number changes involved in the pathogenesis and development of OS have been detected by NGS approaches^31^. Recently, we reviewed the top 10 frequently mutated genes (e.g., TP53, RB1, PTEN, DLG2, MYC, ATRX, NF1, CCNE1, CDKN2A, and PIK3CA) and some tumor‐specific CNAs (e.g., MYC, CCNE1, VEGFA, BRCA1/2, TP53, RB1, CDKN2A/2B) in OS tissues identified by NGS technology^32^. More recently, Mirabello and her colleagues found that a higher‐than‐expected frequency of pathogenic or likely pathogenic germline variants existed in genes not previously linked to OS (e.g., CDKN2A, MEN1, VHL, POT1, APC, MSH2, and ATRX)^33^. Furthermore, some studies have indicated that familial occurrence of OS may present an inherited genetic predisposition to this tumor^19^, ^34^. Several genetic variants or molecular abnormalities have been identified to be associated with the cooccurrence of OS in siblings, such as germline mutation of RB1, TP53, or ATRX genes^8^, ^24^, loss of heterozygosity of RB1 and TP53^24^, 13;14 chromosomal rearrangement^19^, HLA phenotypes^15^, ^21^, and RECQL4 mutation^25^. The occurrence of OS in identical twins is a relatively rare event but may present the best opportunity to understand the genetic factors and molecular mechanisms underlying the tumorigenesis and progression of this disease in humans^4^. Therefore, it is necessary to perform NGS for identical twins in the following study.

However, the duration of follow‐up was relatively short in the present study. A longer time follow‐up and regular imaging evaluation are needed to confirm whether the identical twin of these patients will suffer OS in the future.

## Authorship Declaration

All authors listed meet the authorship criteria according to the latest guidelines of the International Committee of Medical Journal Editors, and they are all in agreement with the manuscript.

## Availability of Data and Materials

All data generated or analyzed during this study are included in this published article.

## Ethics Approval and Consent to Participate

Our study was approved by the Ethics Committee of the PLA 960th hospital. All adult patients and parents for children who participate in the study provided written informed consent. A copy of the consent form is available for review.

## Patient Consent for Publication

The patient provided written informed consent for the publication of associated data and accompanying images.
